# Creating a sustainable brain health navigator model to improve the diagnostic journey for Alzheimer's disease: the experience of one health care system in Michigan

**DOI:** 10.1002/alz70860_101272

**Published:** 2025-12-23

**Authors:** Cara Leahy, Phincy Arangattu

**Affiliations:** ^1^ Memorial Healthcare Institute for Neuroscience, Owosso, MI, USA

## Abstract

**Background:**

Memorial Healthcare is an independent community hospital established in 1921. Memorial Healthcare is integrated with Memorial Medical Associates, which includes over 100 employed physicians and 30 primary care providers across eight cities in Michigan. Memorial Healthcare received a 4‐star rating from the Centers for Medicare & Medicaid Services in 2024 and is the sole hospital provider for residents of Shiawassee County. The next closest option for patients is over 20 miles away. There is a crucial care shortage for the aging population, and there are not enough care facilities specializing in memory care nor enough neurologists in the state to handle the treatment demand.

**Method:**

The pilot program will be conducted at a rural clinic, the King Street Family Medicine in Owosso, Michigan. The primary care site is located across the parking lot from the Institute for Neuroscience, the main Hospital, lab services, and imaging. Besides being connected geographically, all providers are connected quarterly through MMA and meet monthly through departmental and clinic meetings. Patient navigator programs are vital to the continuity of care throughout Memorial Healthcare. The BHN Program will help educate and support primary care physicians and medical assistants will be taught when and how to test for MCI which will help assessment of symptoms. Helping primary care physicians understand the neurologic referral process will aid in a more streamlined workflow. Overall, it will provide a more timely and accurate diagnosis for patients and families.

**Result:**

Memorial anticipates significant improvements in the early detection of cognitive impairment. The collaboration between primary care providers and the Memory Care Clinic is expected to deliver a more streamlined referral process. Patient experiences will improve as transitions from primary care to specialty care become smoother and more efficient. Access to treatments will expand as workflows are optimized, and community partnerships will help address social determinants of health such as transportation and food insecurity.

**Conclusion:**

The Brain Health Navigator Model and other resources of DAC‐SP support the implementation of boundary spanning roles across primary and specialty care, designed for a timely and accurate diagnostic journey across Memorial Healthcare and similar health systems.

